# Supplementary material for: AAA+ protease-adaptor structures reveal altered conformations and ring specialization
Source: Nat Struct Mol Biol. 2022 Nov 3;29(11):1068–79. doi: 10.1038/s41594-022-00850-3 (PMC9663308; doi:10.1038/s41594-022-00850-3)
Supplement: Supplementary file 1 — Supplementary Table 1, Figs. 1–7, references and unprocessed gel image for Supplementary Fig. 1a. [file 41594_2022_850_MOESM1_ESM.pdf]

nature structural & molecular biology

Article https://doi.org/10.1038/s41594-022-00850-3

AAA+ protease-adaptor structures reveal altered conformations and ring specialization

In the format provided by the authors and unedited

SUPPLEMENTARY INFORMATION

Supplementary Table 1. Oligonucleotides used in this study.

Oligonucleotide Name	Purpose	Oligonucleotide Sequence (5′ to 3′)
D1 pore-2 QTQ For	Round-the-horn mutagenesis of ^{QTQ}ClpA	CAAGGTGGTCAGGTCGATGCG
D1 pore-2 QTQ Rev	Round-the-horn mutagenesis of ^{QTQ}ClpA	AGTCTGACCCGCACCGATAATGG
D1 pore-2 G For	Round-the-horn mutagenesis of ^{GGG}ClpA	GGAGGTGGTCAGGTCGATGCG
D1 pore-2 GG Rev	Round-the-horn mutagenesis of ^{GGG}ClpA	GCCTCCACCCGCACCGATAATGG
D1 pore-2 298 For	Round-the-horn mutagenesis of $^{\Delta 295\text{-}297}$ClpA and ^{KYR}ClpA	GGTGGTCAGGTCGATGCG
D1 pore-2 294 Rev	Round-the-horn mutagenesis of $^{\Delta 295\text{-}297}$ClpA	ACCCGCACCGATAATGG
D1 pore-2 KYR Rev	Round-the-horn mutagenesis of ^{KYR}ClpA	ACGATATTTACCCGCACCGATAATGG

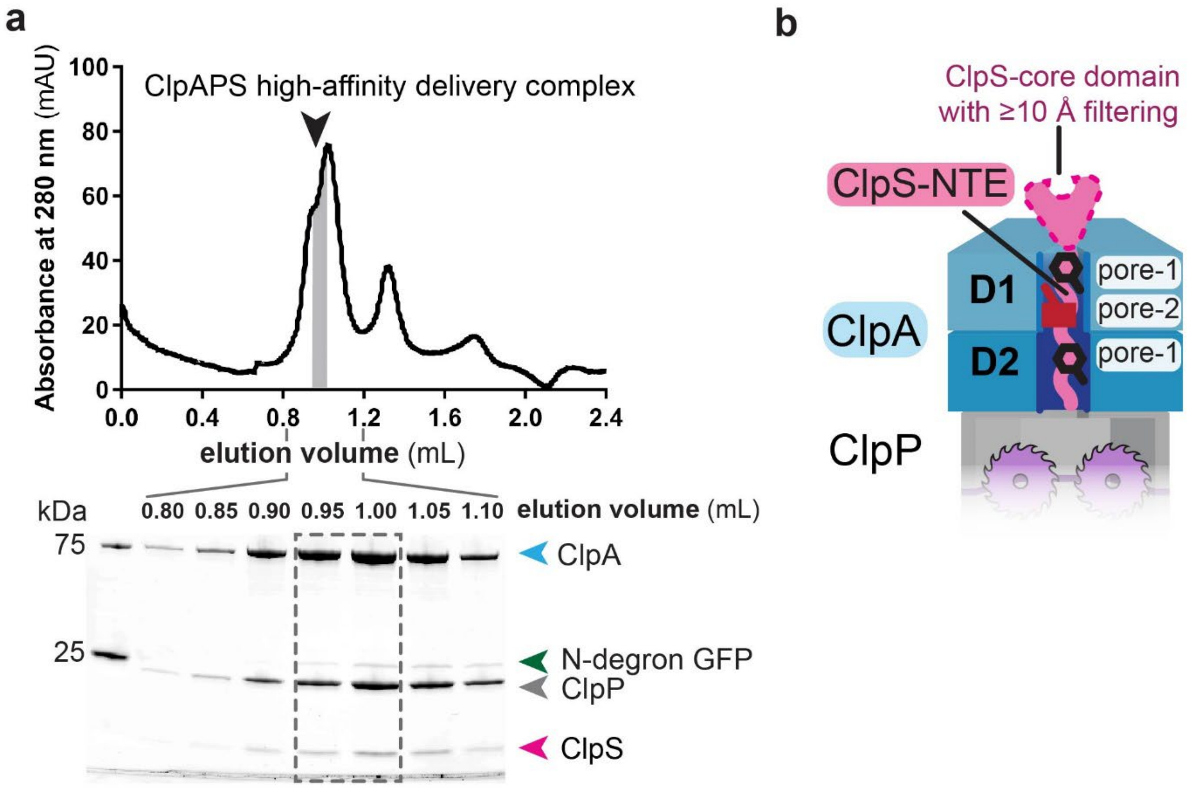

Supplementary Figure 1. Overview of ClpAPS cryo-EM structural features.
a, Size-exclusion chromatography (top panel) of a complex of ClpA, ClpP, ClpS, and YLFVQELA-GFP (an N-degron substrate) assayed by SDS-PAGE (bottom panel). Gray shaded area and boxed area indicate the fractions pooled for cryo-EM. Preparation of ClpAPS•YLFVQELA-GFP complexes by SEC was performed independently five times (during initial grid screening and optimization); only the shown SEC-purified sample was used to prepare the single grid imaged for data collection. Uncropped gel image at the end of the SI file. **b**, Cartoon of pore loops that interact with the ClpS NTE and proteins resolved in cryo-EM structures.

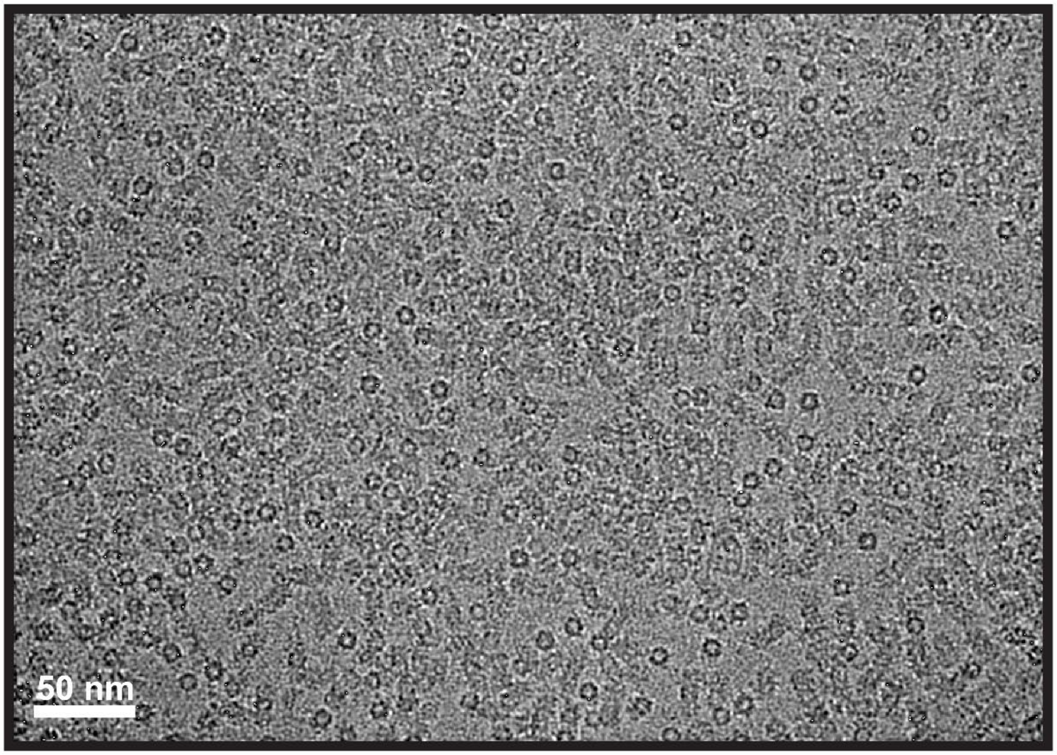

Supplementary Figure 2. ClpAPS cryo-EM micrograph.
Representative cryo-EM micrograph of ClpAPS•N-degron GFP complexes from cryo-EM data collection (9,169 micrographs collected total). The scale bar corresponds to 50 nm. The majority of particles are doubly capped with two $ClpA_6$ molecules per $ClpP_{14}$.

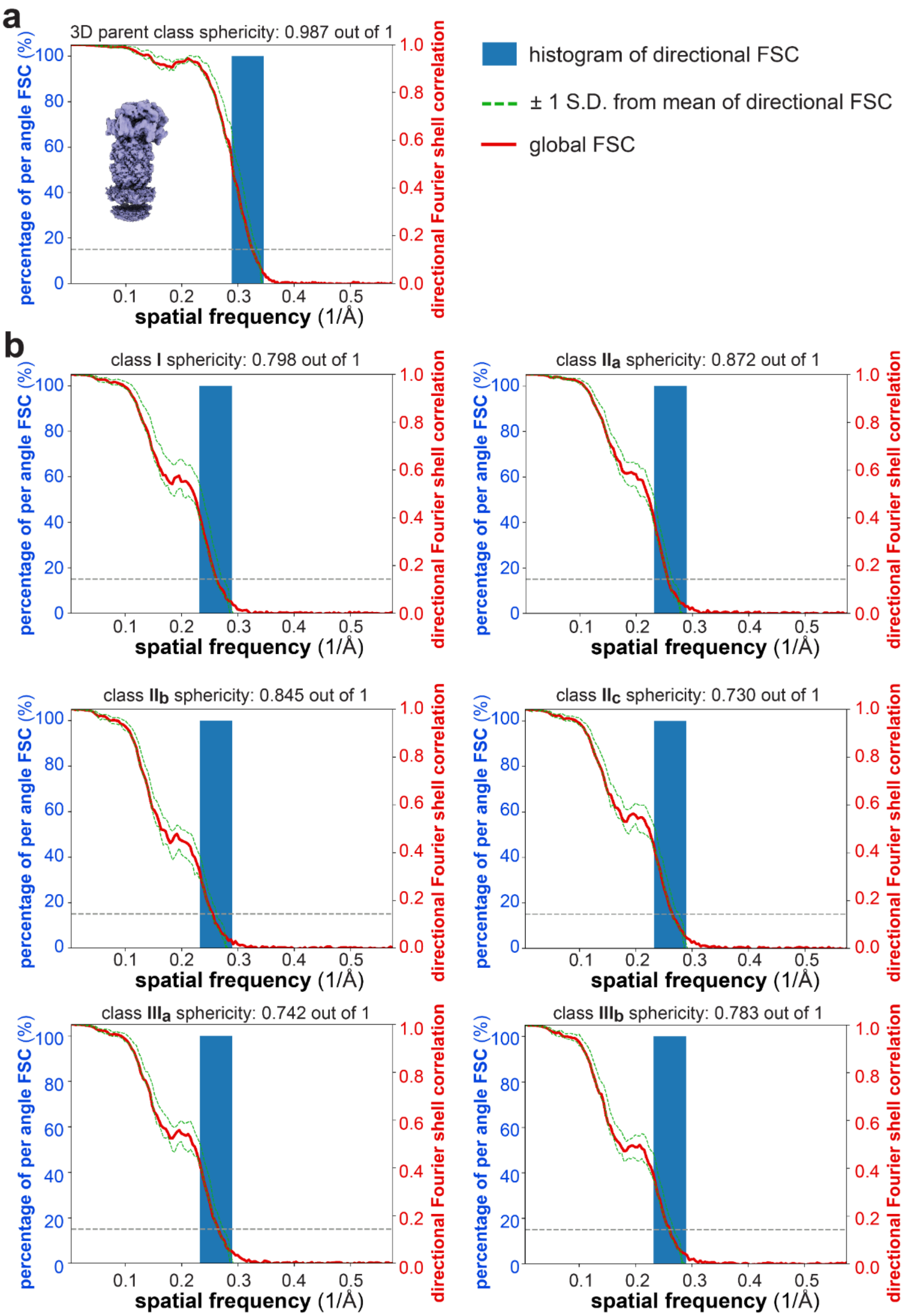

Supplementary Figure 3. 3DFSC sphericity

3DFSC plots of classes **I**, **II**, and **III** and parent class prior to focused classification. Due to non-uniform box dimensions following density modification, intermediate maps from RELION-3 (from 3D auto-refine) were used as 3DFSC inputs for class **I**, **II**, and **III** structures.

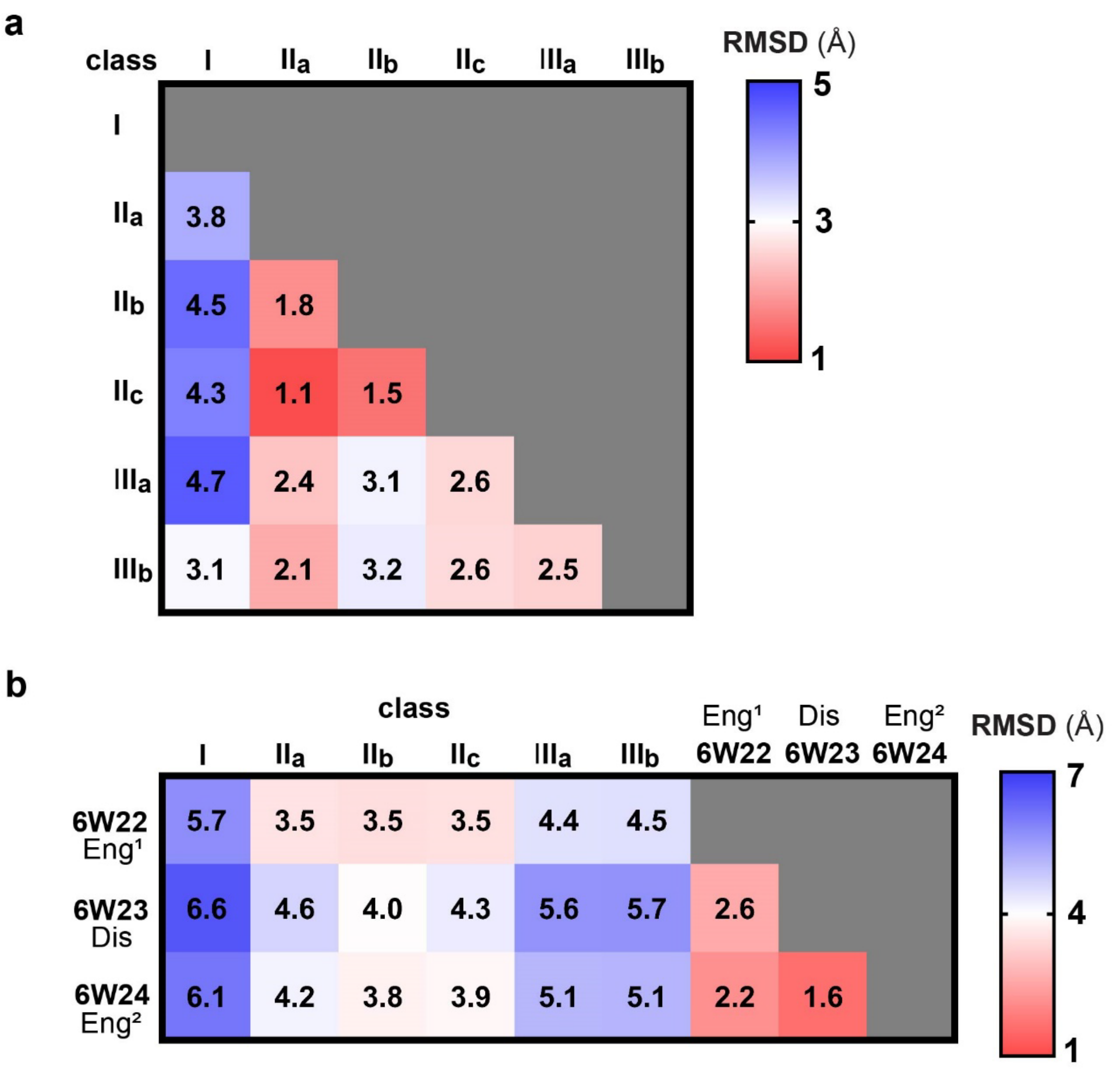

Supplementary Figure 4. Cα RMSD analysis of ClpAP structures.
a, Pairwise RMSD (Å) calculations of class **I**, **II**, and **III** subclasses between ClpA Cα atoms in subunits A through F using PyMOL "align". **b**, Pairwise RMSD comparisons of ClpA Cα atoms in ClpAPS cryo-EM structures and ClpAP•RepA-GFP structures (PDB 6W22, 6W23, and 6W24), using PyMOL "align".

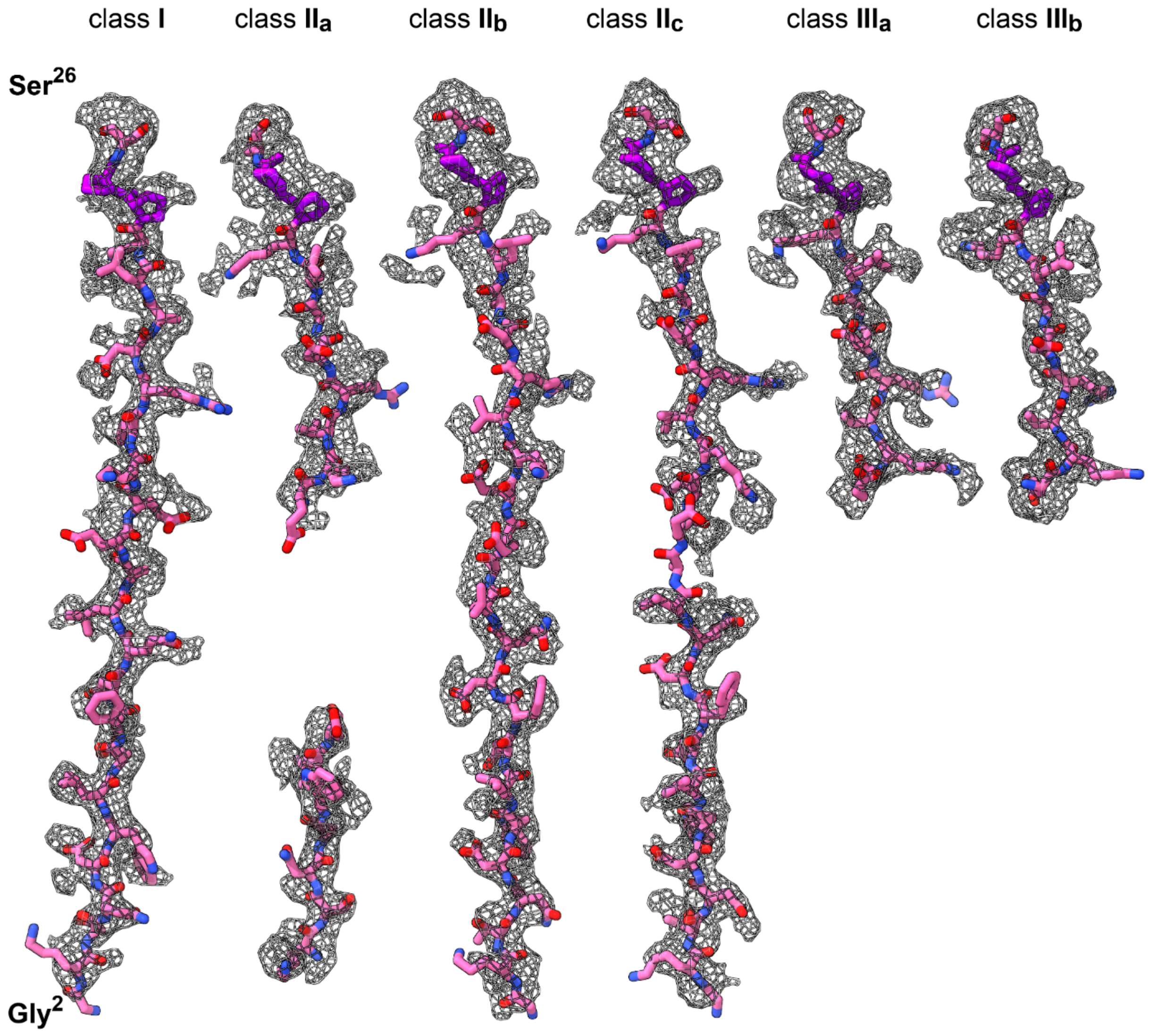

Supplementary Figure 5. ClpS NTE map–model comparisons.
ClpS NTE density (mesh at contour level 0.25) and atomic models (shown in sticks) in the ClpA axial channel in classes **I**, **II**, and **III**. The junction sequence residues, Pro^{24} and Pro^{25}, are colored purple, whereas the rest of the NTE is colored pink.

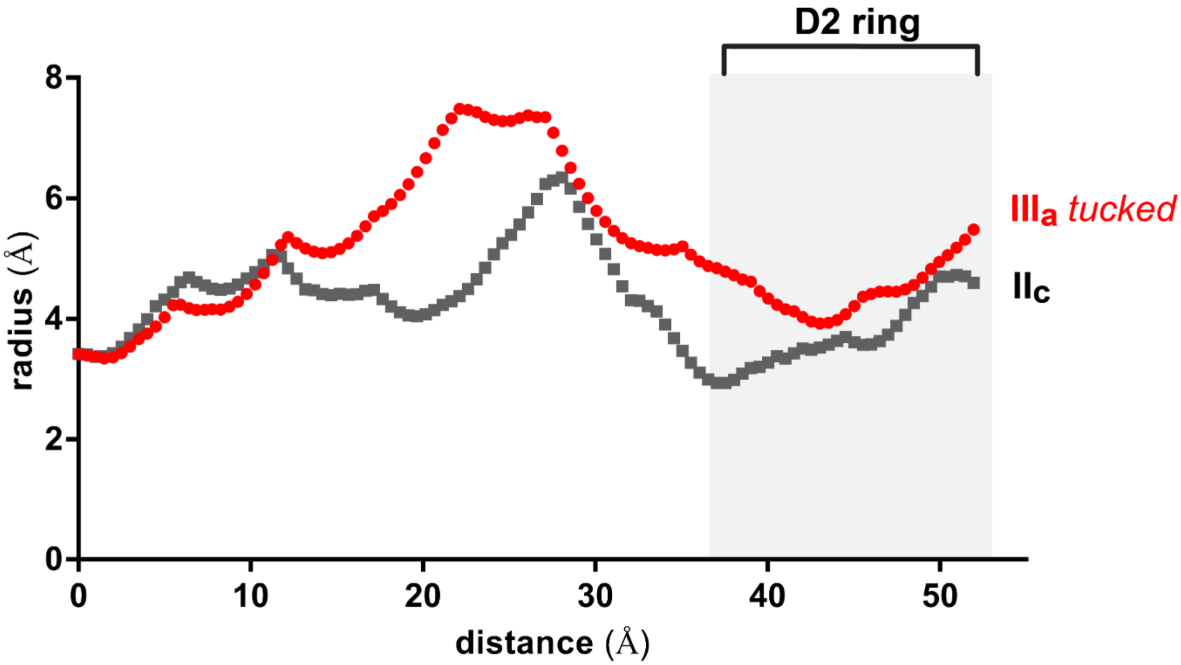

Supplementary Figure 6. Axial channel radius comparison. Plot of channel radius vs. channel distance (length of channel from top to bottom of ClpA) calculated by CAVER[1] in class-**II**$_c$ (dark gray squares) and class-**III**$_a$ (red circles) atomic models. Portion of axial channel in D2 ring is indicated by the light gray box.

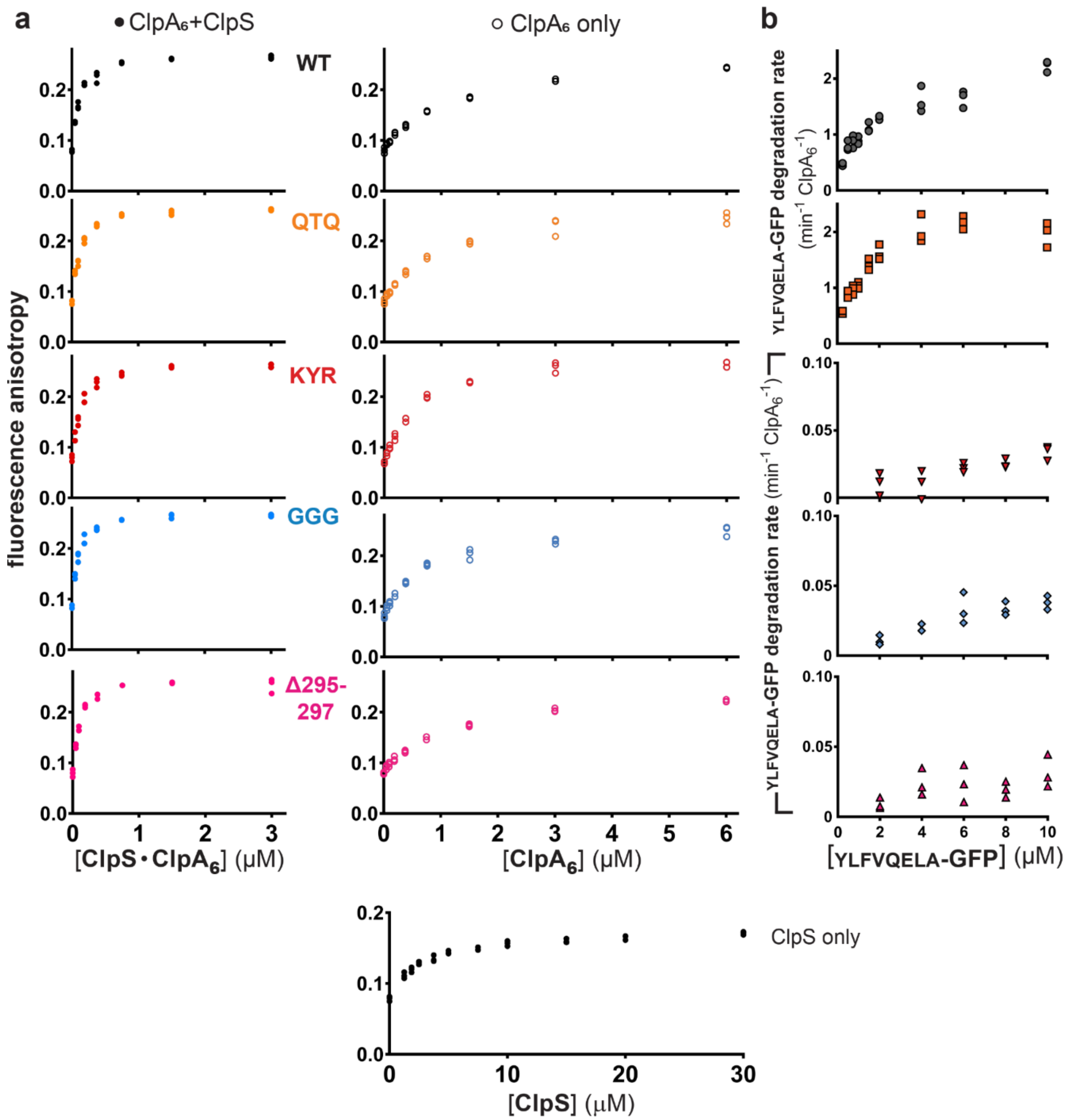

Supplementary Figure 7. Individual fluorescence anisotropy and YLFVQELA-GFP degradation rate values from Fig. 5a–b. a, Fluorescence anisotropy of ClpA pore-2 variants alone ($ClpA_6$, open circles), with an equimolar mixture with ClpS (+ClpS, filled circles), or ClpS only (bottom right panel), titrated in increasing concentrations against a fixed concentration of fluorescein-labeled N-degron peptide. **b**, Kinetic analysis of YLFVQELA-GFP degradation by ClpA D1 pore-2 variants (see *Methods* for concentrations). The graphed y-axis of WT and the KYR variant ranges from 0–2.5 min^{-1} $ClpA_6^{-1}$, whereas the y-axis shown for KYR, GGG, and Δ295–297 variants ranges from 0–0.10 min^{-1} $ClpA_6^{-1}$.

SUPPLEMENTARY REFERENCES

1. Jurcik, A., Bednar, D., Byska, J., Marques, S. M., Furmanova, K., Daniel, L., Kokkonen, P., Brezovsky, J., Strnad, O., Stourac, J., Pavelka, A., Manak, M., Damborsky, J., & Kozlikova, B. CAVER Analyst 2.0: analysis and visualization of channels and tunnels in protein structures and molecular dynamics trajectories. Bioinformatics (Oxford, England), 34(20), 3586–3588 (2018).

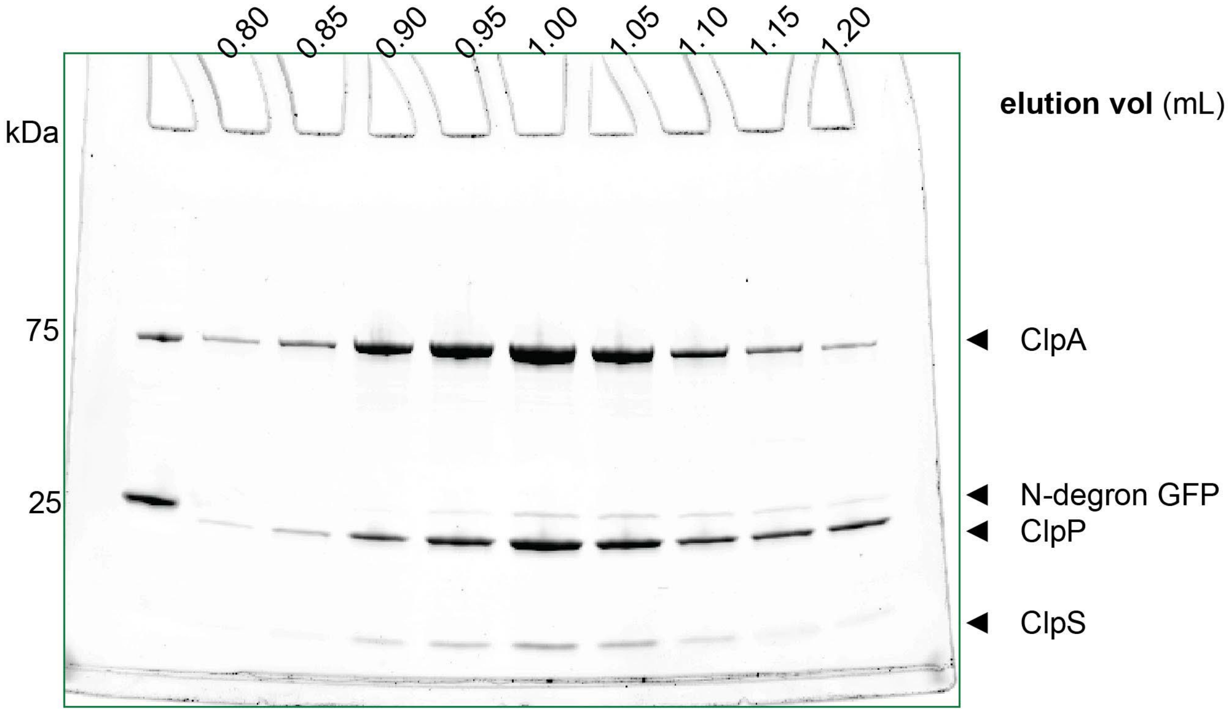

Unprocessed gel image for Supplementary Fig. 1a. SDS-PAGE gel (stained with SYPRO Red) of ClpAPS•YLFVQELA-GFP complexes prepared by SEC.
