# Supplementary material for: AAA+ protease-adaptor structures reveal altered conformations and ring specialization
Source: Nat Struct Mol Biol. 2022 Nov 3;29(11):1068–79. doi: 10.1038/s41594-022-00850-3 (PMC9663308; doi:10.1038/s41594-022-00850-3)
Supplement: Supplementary file 2 — Reporting Summary [file 41594_2022_850_MOESM2_ESM.pdf]

nature portfolio

Corresponding author(s): Tania A. Baker

Last updated by author(s): 2022-8-24

Reporting Summary

Nature Portfolio wishes to improve the reproducibility of the work that we publish. This form provides structure for consistency and transparency in reporting. For further information on Nature Portfolio policies, see our Editorial Policies and the Editorial Policy Checklist.

Statistics

For all statistical analyses, confirm that the following items are present in the figure legend, table legend, main text, or Methods section.

n/a	Confirmed	
☐	☒	The exact sample size (*n*) for each experimental group/condition, given as a discrete number and unit of measurement
☐	☒	A statement on whether measurements were taken from distinct samples or whether the same sample was measured repeatedly
☒	☐	The statistical test(s) used AND whether they are one- or two-sided<br>*Only common tests should be described solely by name; describe more complex techniques in the Methods section.*
☒	☐	A description of all covariates tested
☒	☐	A description of any assumptions or corrections, such as tests of normality and adjustment for multiple comparisons
☐	☒	A full description of the statistical parameters including central tendency (e.g. means) or other basic estimates (e.g. regression coefficient) AND variation (e.g. standard deviation) or associated estimates of uncertainty (e.g. confidence intervals)
☒	☐	For null hypothesis testing, the test statistic (e.g. *F*, *t*, *r*) with confidence intervals, effect sizes, degrees of freedom and *P* value noted<br>*Give P values as exact values whenever suitable.*
☒	☐	For Bayesian analysis, information on the choice of priors and Markov chain Monte Carlo settings
☒	☐	For hierarchical and complex designs, identification of the appropriate level for tests and full reporting of outcomes
☒	☐	Estimates of effect sizes (e.g. Cohen's *d*, Pearson's *r*), indicating how they were calculated

Our web collection on statistics for biologists contains articles on many of the points above.

Software and code

Policy information about availability of computer code

Data collection	Data collection was performed using SerialEM 3.6.
Data analysis	RELION 3.0.8, MotionCor2 1.30, and CTFFIND4 4.1.13 were used for cryo-EM data processing. Final maps were density-modified and autosharpened in PHENIX 1.20. For molecular modeling, Chimera "fit in map" was used for docking. Real-space refinement was performed using PHENIX 1.20, and model building was performed in Coot 0.9.8. MolProbity was used to evaluate geometry of the final models. 3DFSC 3.0 was used to evaluate directional resolution. Phenix cryo-EM validation and EMRinger were used to assess model geometry and map quality. Low-pass filtered cryo-EM maps were generated using EMAN2 2.91 "e2proc3d". Figures and movies of cryo-EM structures and models were generated in Chimera 1.14, ChimeraX 1.2.5, and PyMOL 2.3 (Schrodinger, LLC).<br><br>Buried surface area calculations were performed using the EBI PISA webserver. Axial channel diameter and length were measured using CAVER.<br><br>Amino-acid sequences of E. coli ClpA, ClpB, and ClpC proteins were aligned using MUSCLE with MEGA7, followed by visualization in Jalview 1.8.<br><br>Biochemical experiments were analyzed in GraphPad Prism 7.

For manuscripts utilizing custom algorithms or software that are central to the research but not yet described in published literature, software must be made available to editors and reviewers. We strongly encourage code deposition in a community repository (e.g. GitHub). See the Nature Portfolio guidelines for submitting code & software for further information.

Data

Policy information about availability of data

All manuscripts must include a data availability statement. This statement should provide the following information, where applicable:

- Accession codes, unique identifiers, or web links for publicly available datasets
- A description of any restrictions on data availability
- For clinical datasets or third party data, please ensure that the statement adheres to our policy

Maps were deposited in the Electron Microscopy Data Bank (EMDB) under accession codes: EMD-26556 for class I, EMD-26554 for class IIa, EMD-26555 for class IIb, EMD-26558 for class IIc, EMD-26557 for class IIIa, EMD-26559 for class IIIb. Atomic models were deposited in the Protein Data Bank (PDB) under accession codes: 7UIX for class I, 7UIV for class IIa, 7UIW for class IIb, 7UIZ for class IIc, 7UIY for class IIIa, and 7UJ0 for class IIIb.

Amino-acid sequences of ClpA, ClpB, and ClpC proteins were downloaded from UniProtKB (also provided in Fig. 4 and Ext. Data Fig. 8): E. coli ClpA (P0ABH9), V. cholerae ClpA (Q9KSW2), P. aeruginosa ClpA (Q9I0L8), C. acetobutylicum ClpA (Q97I30), C. vibriodes ClpA (Q9A5H9), B. diazoefficiens ClpA (Q89JW6), X. fastidiosa ClpA (Q87DL7), N. meningitidis ClpA (Q9JZZ6), D. radiodurans ClpA (Q9RWS7), M. tuberculosis ClpB (P9WPD1), L. interrogans ClpB (Q8F509), E. coli ClpB (P63284), T. thermophilus ClpB (Q9RA63), L. biflexa ClpC (B0SM25), M. tuberculosis ClpC (P9WPC9), and B. subtilis ClpC (P37571). Previously published atomic models are available from the PDB: E. coli ClpS (PDB 3O2B) and for E. coli ClpAP•RepA-GFP Eng1 (PDB 6W22), Dis (PDB 6W23), and Eng2 (PDB 6W24).

The uncropped gel shown in Supplementary Figure 1a is available in the Supplementary Information file. The values plotted in Fig. 5 and Ext. Data Fig. 10 are provided as Source Data online. Any additional information required to reanalyze the data reported in this study is available from the corresponding author (tabaker@mit.edu).

Human research participants

Policy information about studies involving human research participants and Sex and Gender in Research.

Reporting on sex and gender	N/A
Population characteristics	N/A
Recruitment	N/A
Ethics oversight	N/A

Note that full information on the approval of the study protocol must also be provided in the manuscript.

Field-specific reporting

Please select the one below that is the best fit for your research. If you are not sure, read the appropriate sections before making your selection.

☒ Life sciences ☐ Behavioural & social sciences ☐ Ecological, evolutionary & environmental sciences

For a reference copy of the document with all sections, see nature.com/documents/nr-reporting-summary-flat.pdf

Life sciences study design

All studies must disclose on these points even when the disclosure is negative.

Sample size	For biochemical experiments, n greater than or equal to 3 is sufficient, as each reaction contains thousands of molecules. The method of computing sample size for biochemical experiments is not applicable and therefore not performed, as performing biochemical experiments in biological triplicate is a widely-used replication standard.<br><br>For cryo-EM data-processing, all classes exceeded 30,000 particles.<br><br>For both biochemical experiments and cryo-EM structures, the sample sizes correspond to those previously published in our field and other structure-function studies.
Data exclusions	no data exclusions were used
Replication	Each biochemical assay was performed in at least triplicate. ClpA variants were purified and tested at least two times to ensure the activity between preps were consistent. Assay results of wild-type ClpA was benchmarked against assays with other batches of wild-type ClpA.<br><br>ClpAPS cryo-EM classes and atomic models were compared to those of a previous dataset (Lopez et. al 2020) for verification of the data processing methods used.

	All attempts at replication were successful.
Randomization	n/a; we did not randomize samples, as this does not apply to our in vitro studies. In essence, each aliquot of protein added is a random sample of thousands of molecules, as we cannot select individual molecules in the bulk biochemical assays used in this study. No experiments presented in this work required randomization to obtain biologically meaningful and significant results.
Blinding	n/a; we did not perform blinding, as this does not apply to our in vitro studies. In bulk biochemical studies, we cannot identify individual molecules to select them for analysis in our experiments. It is not possible to blind the data in this study.

Reporting for specific materials, systems and methods

We require information from authors about some types of materials, experimental systems and methods used in many studies. Here, indicate whether each material, system or method listed is relevant to your study. If you are not sure if a list item applies to your research, read the appropriate section before selecting a response.

Materials & experimental systems

n/a	Involved in the study
☒	☐ Antibodies
☒	☐ Eukaryotic cell lines
☒	☐ Palaeontology and archaeology
☒	☐ Animals and other organisms
☒	☐ Clinical data
☒	☐ Dual use research of concern

Methods

n/a	Involved in the study
☒	☐ ChIP-seq
☒	☐ Flow cytometry
☒	☐ MRI-based neuroimaging
